# Supplementary material for: Measuring four facets of emotion beliefs in Germany: A German-language adaptation of the EBQ and its comparability across gender and different emotion abilities
Source: PLoS One. 2025 Jan 2;20(1):e0316007. doi: 10.1371/journal.pone.0316007 (PMC11694981; doi:10.1371/journal.pone.0316007)
Supplement: S1 Table — (PDF) [file pone.0316007.s001.pdf]

S1 Table

Intercorrelations of the 16 single items of the EBQ

Variable	1	2	3	4	5	6	7	8	9	10	11	12	13	14	15	16
EBQ-Item 2	.42	—														
EBQ-Item 3	.20	.18	—													
EBQ-Item 4	.15	.05	.21	—												
EBQ-Item 5	.61	.39	.25	.20	—											
EBQ-Item 6	.12	.19	.15	.24	.20	—										
EBQ-Item 7	.16	.12	.41	.30	.28	.20	—									
EBQ-Item 8	.21	.09	.14	.70	.24	.13	.37	—								
EBQ-Item 9	.45	.43	.25	.17	.51	.22	.25	.19	—							
EBQ-Item 10	.38	.49	.28	.23	.49	.32	.22	.20	.43	—						
EBQ-Item 11	.17	.13	.23	.15	.28	.12	.32	.18	.32	.10	—					
EBQ-Item 12	.26	.10	.09	.47	.23	.14	.25	.58	.10	.11	.12	—				
EBQ-Item 13	.24	.29	.06	.14	.30	.47	.25	.16	.33	.33	.15	.22	—			
EBQ-Item 14	.29	.38	.24	.17	.41	.25	.28	.23	.32	.51	.27	.23	.34	—		
EBQ-Item 15	.20	.24	.34	.15	.27	.10	.41	.20	.32	.20	.67	.11	.17	.30	—	
EBQ-Item 16	.09	.07	.12	.45	.22	.24	.29	.43	.22	.14	.24	.34	.12	.23	.17	—
