# Supplementary material for: Measuring four facets of emotion beliefs in Germany: A German-language adaptation of the EBQ and its comparability across gender and different emotion abilities
Source: PLoS One. 2025 Jan 2;20(1):e0316007. doi: 10.1371/journal.pone.0316007 (PMC11694981; doi:10.1371/journal.pone.0316007)
Supplement: S2 Table — (PDF) [file pone.0316007.s002.pdf]

S2 Table

Intercorrelations of the four factors of EBQ, namely, controllability and usefulness of negative and positive emotions

**Variable**	**EBQ-C-N**	**EBQ-C-P**	**EBQ-U-N**	**EBQ-U-P**
Controllability (negative)	—			
Controllability (positive)	.62	—		
Usefulness (negative)	.42	.34	—	
Usefulness (positive)	.30	.39	.31	—
