# Supplementary material for: Measuring four facets of emotion beliefs in Germany: A German-language adaptation of the EBQ and its comparability across gender and different emotion abilities
Source: PLoS One. 2025 Jan 2;20(1):e0316007. doi: 10.1371/journal.pone.0316007 (PMC11694981; doi:10.1371/journal.pone.0316007)
Supplement: S3 Table — (PDF) [file pone.0316007.s003.pdf]

S3 Table

Test of measurement variance across low and high emotional reactivity in respondents for the whole emotion belief model and its four facets

**Models**		**Configural**	**Metric**	**Scalar**	**Strict**
Complete 4-factor model	χ^2	289.36	307.49	319.77	352.28
	df	194	206	218	234
	p	< .001	< .001	< .001	< .001
	CFI	.903	.897	.897	.880
	RMSEA (90% C.I.)	.075 [.056; .092]	.075 [.057; .091]	.073 [.055; .089]	.076 .059; .091]
	SRMR	.075	.084	.087	.090
	ΔCFI		.006	< .001	.017
	ΔRMSEA		< .001	.002	.003
	ΔSRMR		.009	.003	.003
	Decision		Accept	Accept	**Reject**
Controllability (negative emotions)	χ^2	8.16	9.54	12.17	18.45
	df	4	7	10	14
	p	.086	.216	.274	.187
	CFI	.980	.988	.989	.978
	RMSEA (90% C.I.)	.095 [.000; .188]	.056 [.000; .135]	.043 [.000; .114]	.052 [.000; .110]
	SRMR	.034	.042	.049	.054
	ΔCFI		.008	.001	.011
	ΔRMSEA		.039	.013	.009
	ΔSRMR		.008	.007	.005
	Decision		Accept	Accept	**Accept**
Controllability (positive emotions)	χ^2	1.35	6.72	11.70	14.98
	df	4	7	10	14
	p	.854	.459	.306	.379
	CFI	1.000	1.000	.984	.991

**Models**		**Configural**	**Metric**	**Scalar**	**Strict**
	RMSEA<br>(90% C.I.)	.000<br>[.000; .087]	.000<br>[.000; .127]	.044<br>[.000; .128]	.028<br>[.000; .109]
	SRMR	.014	.054	.070	.067
	ΔCFI		< .001	.016	.007
	ΔRMSEA		< .001	.044	.016
	ΔSRMR		.040	.016	.003
	Decision		Accept	Accept	**Accept**
Usefulness<br>(negative emotions)	χ^2	6.40	8.17	8.57	9.95
	df	2	5	8	12
	p	.041	.147	.380	.620
	CFI	.981	.986	.998	1.000
	RMSEA<br>(90% C.I.)	.137<br>[.024; .263]	.074<br>[.000; .161]	.025<br>[.000; .113]	.000<br>[.000; .081]
	SRMR	.023	.031	.032	.042
	ΔCFI		.005	.012	.002
	ΔRMSEA		.065	.049	.025
	ΔSRMR		.008	.001	.010
	Decision		Accept	Accept	**Accept**
Usefulness<br>(positive emotions)	χ^2	9.58	10.64	11.31	22.88
	df	4	7	10	14
	p	.048	.155	.334	.062
	CFI	.984	.989	.996	.974
	RMSEA<br>(90% C.I.)	.109<br>[.009; .200]	.067<br>[.000; .143]	.034<br>[.000; .109]	.074<br>[.000; .126]
	SRMR	.028	.034	.035	.061
	ΔCFI		.005	.007	.022
	ΔRMSEA		.042	.033	.040
	ΔSRMR		.006	.001	.026
	Decision		Accept	Accept	Accept

4
