# Supplementary material for: Measuring four facets of emotion beliefs in Germany: A German-language adaptation of the EBQ and its comparability across gender and different emotion abilities
Source: PLoS One. 2025 Jan 2;20(1):e0316007. doi: 10.1371/journal.pone.0316007 (PMC11694981; doi:10.1371/journal.pone.0316007)
Supplement: S4 Table — (PDF) [file pone.0316007.s004.pdf]

**S4 Table**

*Test of measurement variance across low and high emotional reactivity self-efficacy in respondents for the whole emotion belief model and its four facets*

| Models | | Configural | Metric | Scalar | Strict |
|---|---|---|---|---|---|
| Complete 4-factor model | $\chi^2$ | 270.64 | 280.01 | 360.33 | 383.02 |
| | *df* | 194 | 206 | 218 | 234 |
| | *p* | < .001 | < .001 | < .001 | < .001 |
| | CFI | .913 | .916 | .839 | .831 |
| | RMSEA (90% C.I.) | .066 [.046; .084] | .063 [.043; .080] | .084 [.069; .100] | .083 [.068; .098] |
| | SRMR | .080 | .085 | .120 | .119 |
| | ΔCFI | | .003 | .077 | .008 |
| | ΔRMSEA | | .003 | .021 | .001 |
| | ΔSRMR | | .005 | .035 | .001 |
| | Decision | | Accept | Reject | **Reject** |
| Controllability (negative emotions) | $\chi^2$ | 3.84 | 9.10 | 9.65 | 21.14 |
| | *df* | 4 | 7 | 10 | 14 |
| | *p* | .428 | .246 | .471 | .098 |
| | CFI | 1.000 | .984 | 1.000 | .945 |
| | RMSEA (90% C.I.) | .000 [.000; .155] | .057 [.000; .149] | .000 [.000; .111.] | .075 [.000; .136] |
| | SRMR | .027 | .058 | .060 | .063 |
| | ΔCFI | | .016 | .016 | .055 |
| | ΔRMSEA | | .057 | .057 | .075 |
| | ΔSRMR | | .031 | .002 | .003 |
| | Decision | | Accept | Accept | **Accept** |
| Controllability (positive emotions) | $\chi^2$ | 2.58 | 3.40 | 80.18 | 85.28 |
| | *df* | 4 | 7 | 10 | 14 |
| | *p* | .630 | .846 | < .001 | < .001 |
| | CFI | 1.000 | 1.000 | .366 | .356 |

| Models | | Configural | Metric | Scalar | Strict |
|---|---|---|---|---|---|
| | RMSEA (90% C.I.) | .000 [.000; .129] | .000 [.000; .073] | .277 [.223; .335] | .236 [.189; .285] |
| | SRMR | .021 | .035 | .251 | .251 |
| | ΔCFI | | < .001 | .644 | .010 |
| | ΔRMSEA | | < .001 | .277 | .041 |
| | ΔSRMR | | .014 | .216 | < .001 |
| | Decision | | Accept | Reject | **Reject** |
| Usefulness (negative emotions) | $\chi^2$ | 0.42 | 1.57 | 3.53 | 6.46 |
| | *df* | 2 | 5 | 8 | 12 |
| | *p* | .809 | .905 | .897 | .891 |
| | CFI | 1.000 | 1.000 | 1.000 | 1.000 |
| | RMSEA (90% C.I.) | .000 [.000; .128] | .000 [.000; .060] | .000 [.000; .054] | .000 [.000; .049] |
| | SRMR | .005 | .022 | .032 | .047 |
| | ΔCFI | | < .001 | < .001 | < .001 |
| | ΔRMSEA | | < .001 | < .001 | < .001 |
| | ΔSRMR | | .017 | .010 | .015 |
| | Decision | | Accept | Accept | **Accept** |
| Usefulness (positive emotions) | $\chi^2$ | 3.69 | 9.32 | 9.90 | 15.15 |
| | *df* | 4 | 7 | 10 | 14 |
| | *p* | .450 | .230 | .449 | .368 |
| | CFI | 1.000 | .990 | 1.000 | .995 |
| | RMSEA (90% C.I.) | .000 [.000; .152] | .060 [.000; .150] | .000 [.000; .113] | .030 [.000; .108] |
| | SRMR | .020 | .055 | .056 | .065 |
| | ΔCFI | | .010 | .010 | .005 |
| | ΔRMSEA | | .060 | .060 | .030 |
| | ΔSRMR | | .035 | .001 | .009 |
| | Decision | | Accept | Accept | Accept |

4
