# Supplementary figures and images for: Measuring four facets of emotion beliefs in Germany: A German-language adaptation of the EBQ and its comparability across gender and different emotion abilities
Source: PLoS One. 2025 Jan 2;20(1):e0316007. doi: 10.1371/journal.pone.0316007 (PMC11694981; doi:10.1371/journal.pone.0316007)

# 1 S1 Figure

## 2 Structural equation model of the whole model of emotion beliefs

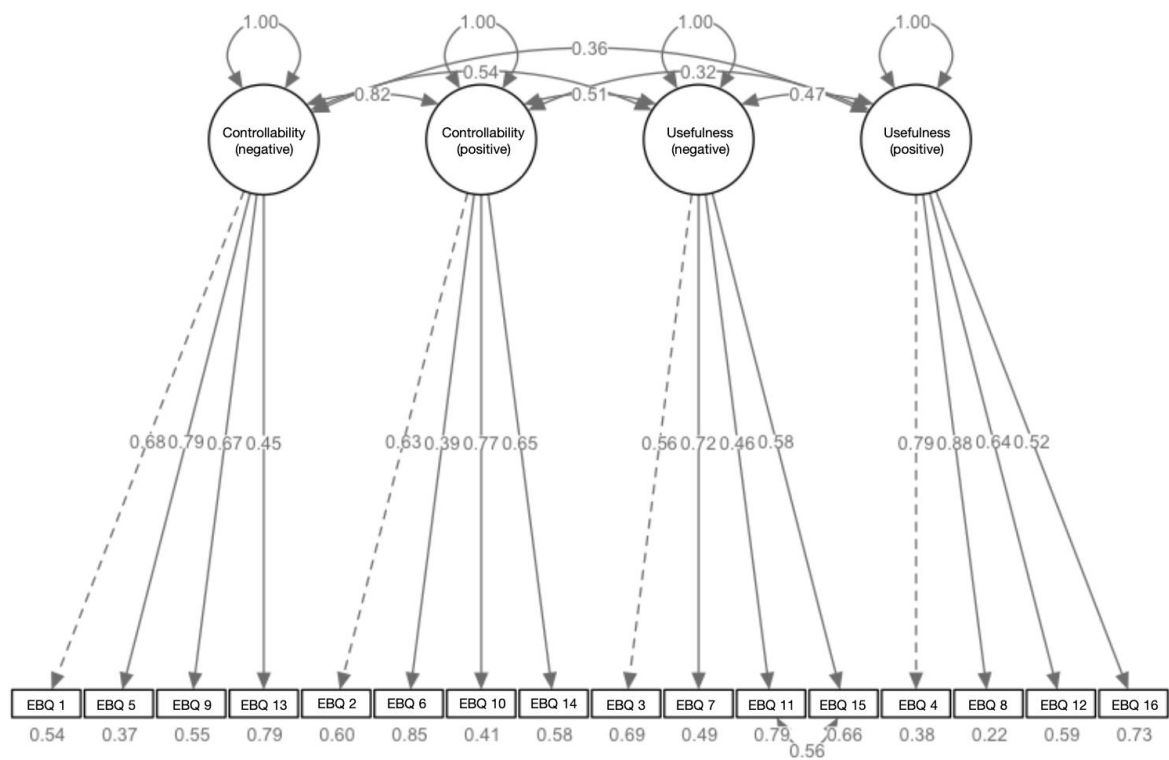

3

Supplement: S1 Fig — (PDF) [file pone.0316007.s005.pdf]
