# Supplementary material for: Measuring four facets of emotion beliefs in Germany: A German-language adaptation of the EBQ and its comparability across gender and different emotion abilities
Source: PLoS One. 2025 Jan 2;20(1):e0316007. doi: 10.1371/journal.pone.0316007 (PMC11694981; doi:10.1371/journal.pone.0316007)
Supplement: Attachment — Submitted filename: Response to Reviewers.docx [file pone.0316007.s006.docx]

**Response to Reviewers**

We thank the reviewers for their helpful comments which allows a higher quality of the manuscript (PONE-D-24-31699). Please find enclosed the revision of our submission to *PLOS ONE*. We have carefully considered each comment and have implemented the suggested changes or responded to comments. In the following sections, we respond on a point-by-point basis (in italic) to the issues raised by the reviewers. We hope that we have dealt satisfactorily with all concerns.

**Reviewer #1:** Thank you for giving me the opportunity to review this study in the field of emotion regulation.

This study presents a lot of valuable work. However, it needs some alterations before it gets published. From my perspective, there is some work to be done and a lot of alterations which although many are managable...
INTRODUCTION
Please, start with a brief review linking otgether all of the variables used in the study.

*We included a brief review linking all relevant variables used in this study.*

Do not use stats in the Introduction and Discussion sections.

*We removed the stats in the Introduction and Discussion section.*

Keep a consistent way in the presentation of findings of previous studies- think of the most appropriate way to present previous studies and stick to it (e.g. refer to specific coefficients, refer to the problematic ones, refer to validity or/and reliability coefficients and any other relevant coefficients…).

*We adjusted parts of the Introduction section.*

The aim of the study to be rewritten in a clear and straightforward way. Mention the contribution of the present study as (a), (b), (c), (d)… after reporting the limitations of previous studies.

*We adjusted our presentation of the aims of the study.*

Factorial Validity: Give more information about “we deem TLI = .883 acceptable”.

*We added following more information about this in L 322-327. At this point, TLI is sensitive to overfit, which is due to complex models that run the risk to overfit the present data. But it is far from any negative value like below .800.*

Measurement invariance: Start this section with the test you use

*We included additional information in L 392-393.*

Discussion: the first paragraph should give the strong findings of the present study. Then you can discuss the findings of your stats analysis in a story telling way.
Each section should start with your findings and then discuss them in relation to other studies e.g. Becerra et al …

*We included a short introduction of the strong findings in the Discussion and adjusted the way, we discussed the findings.*

Check the use of the APA across the document, the tables as well…

***We have checked the document (and tables) for deviations from APA, however, we could not find any substantial ones. Would you mind pointing us to specific lines so that we can adjust the manuscript?***

**Reviewer #2:** In the introduction (L. 105 to 108), the authors summarised the studies validating the EBQ. They neglected to include this study: Kashimura, M., Ishizu, K., & Becerra, R. (2023). Psychometric Examination of the Japanese Version of the Emotion Beliefs Questionnaire 1 2 3 4. Japanese Psychological Research. (Ok, I see now this paper was been brought up in the discussion)Change "...of the below-depicted study" (L.152) to "...of the present study"

*We now included the study by Kashimura et al. (2023) in the introduction.*

At the end of the introduction (L. 153 to 155), the authors state "By using multiple measures to validate the EBQ, we hope to show a broader picture of the EBQ and its abilities to measure beliefs about emotions". Don't say "we hope", just state that you will show. Also, I'm not sure what a "broader picture is". Operationalise this more precisely.

*We rewrote this section (L. 170).*

L175 "(EBQ; (18); German translation: (34))", check this journal and APA guidelines for the use of brackets within brackets.

*We rewrote this section.*

L. 396, change from "For self-efficacy, we only find one difference:..." to "...found..." (past tense).

*We adjusted the expression.*

L. 406 to 409 "a finding that led Becerra et al. (18) to suggest that distinguishing between the two controllability subscales is unnecessary. In our opinion, this assumption is too strong and leads to loss of information. Therefore, we support the examination of all four subscales". Good finding and I agree with the authors' interpretation.

*Thank you!*

L. 460. "The other EBQ subscales did not predict any symptoms". In the context of this section, I'm not sure what this sentence refers to.

*We described these finding in more detail (L. 499).*

Sometimes the subscale "General controllability" is referred to as "General Controllability" and other times as "General Control". Make this consistent.

*We adjusted the expression throughout the manuscript.*

L. 517. "...we found individual with..." change to "...individuals..."

*We adjusted the expression.*

Good job!
