# Supplementary material for: Measuring four facets of emotion beliefs in Germany: A German-language adaptation of the EBQ and its comparability across gender and different emotion abilities
Source: PLoS One. 2025 Jan 2;20(1):e0316007. doi: 10.1371/journal.pone.0316007 (PMC11694981; doi:10.1371/journal.pone.0316007)
Supplement: Attachment — Submitted filename: Response letter to reviewers, PlosOne_December 2024.docx [file pone.0316007.s007.docx]

**Response to Reviewers**

We thank the reviewers for their helpful comments which allows a higher quality of the manuscript (PONE-D-24-31699R). Please find enclosed the revision of our submission to *PLOS ONE*. We have carefully considered each comment and have implemented the suggested changes or responded to comments. In the following sections, we respond on a point-by-point basis (in italic) to the issues raised by the reviewers. We hope that we have dealt satisfactorily with all concerns.

**Reviewer 1**

1. *The authors have taken account of all of my comments and thy seem to have throughtfully revised the paper.*

We very much appreciate this evaluation and thank the reviewer again for their insightful comments in the review process.

**Reviewer 3**

1. *It seems that adolescents (min. age was 14) participated in this study. Did you receive the inform consent from parents in such cases?*

Yes we did, as mentioned in lines 177-178: “In the case of underage participants, the

written consent of their legal guardians was obtained.”

2. *Statistical programs and their versions with corresponding analyses should be indicated.*

We have now included the statistical program used (i.e., R), its version, and indicated that all analyses were done in R (p. 12): “All analyses were conducted in the statistical language R, version 4.3.2.”

3. *Table 2: TLI values in some cases are above 1, and RMSEA values are 0. Please explain such values.*

For more complex models TLIs > 1.00 can point to misspecification and/or overfitting. As it is occurring here, it’s not uncommon, though, for small item sets with simple (i.e., one-factor) models like the structure we usually encounter for subscales exactly as the ones presented in the present mansucript. We now added a note on this in the revised manuscript (p. 16):

“We note that a TLI above 1.00 is not uncommon for small item sets and simple models, which subscales tend to be (e.g., Goretzko et al., 2023; Schermelleh-Engel et al., 2003). Given that it was developed by Tucker and Lewis (1973) specifically as a Nonnormed Fit Index (NNFI), values above 1.00 are not implausible.”

We thank the reviewer for noting the indication of RMSEA being exactly 0. We revised this to now indicate RMSEA < .001, as the R-analysis has indicated it as 0 due to simplifying after three decimal points.

4. *Overall, what is the reason of testing separate subscales with CFA? It is uncommon practice.*

The idea behind testing the fit of independent factors besides testing the whole model is to test if the different factors fall apart if they are not contrasted with the other factors in one model (i.e., dissimilarity in responses to the other items). To use the different subscales of the model independently, their model fit has to be evaluated independently. We have added a note on this to the revised manuscript (p. 15 to 16): “In addition, we tested the model fit of the four subscales independently, in case researchers want to assess a selected subfactor independently (see for another example, Grüning & Lechner, 2022). This can be understood as an extension of why researchers also consider the internal consistency of subscales, testing whether items are not only acceptably similar, but also load unidimensional.”

In this paragraph, we now also revised “factors” to “subscales” to further make clear what the intention of single subscale model fit testing is.

5. *Please test all the models used in Becerra et al. Please examine whether the 4-factor solution is the best one across all possible solutions presented in the original and other validation studies.*

We have now also tested all alternative models by Becerra et al. (2018) showing that the four-factor model shows the best fit across all of them. Thank you very much for this critical suggestion which added substantial support to the proposed four-factor model of EBQ. We have added the results of these analyses to Table 2 and commented on the comparisons in the revised manuscript (p. 15): "To further support the model’s fit, we compared it with the fit of all six alternative models proposed and tested by Becerra et al. (18). The four-factor model showed the best fit in this array of alternative models, fitting the data significantly better than the second best model, *Δx^2^* = 23.44, *p* < .001, *ΔAIC* = 17, *ΔBIC* = 6).”
